# Frequency of thyroid function tests and examinations in participants of a population-based study

**DOI:** 10.1186/s12913-020-4910-7

**Published:** 2020-01-30

**Authors:** Simone Kiel, Till Ittermann, Henry Völzke, Jean-François Chenot, Aniela Angelow

**Affiliations:** 1 Department of General Practice, Institute for Community Medicine, University Medicine Greifswald, Fleischmannstraße 6, 17475 Greifswald, Germany; 2 Department of SHIP/ Clinical-Epidemiological Research, Institute for Community Medicine, University Medicine Greifswald, Greifswald, Germany

**Keywords:** Thyroid disorders, Patient care, Health claims data, Data linkage

## Abstract

**Background:**

Thyroid disorders are common in the adult German population. Little is known about guideline implementation in clinical practice and the prevalence of diagnostic procedures in ambulatory care. The study aims to investigate the use of thyroid hormone measurements, thyroid ultrasound, thyroid scintiscan and associated costs in ambulatory care at population level.

**Methods:**

Data were derived from two independent population-based cohorts of the Study of Health In Pomerania (SHIP). Ambulatory billing data from the Association of Statutory Health Insurance Physicians Mecklenburg-Vorpommern were individually linked for the period 2002–2016 with SHIP data. The main outcomes were the frequency of outpatient ultrasound, scintiscan, serum TSH level measurement, free triiodothyronine (fT3) and free thyroxine (fT4) measurement, TSH-receptor-antibodies and microsomal antibodies measurement within 1 year and 3 years prior to the study entrance of the participants. Multinomial logistic regression models were used to assess the association of age, sex, thyroid medication intake and Charlson-Comorbidity-Index with frequency of TSH measurements and ultrasound examinations.

**Results:**

A total of 5552 participants (47% male, median age 55) were included in the analysis. 25% (1409/5552) had a diagnosed thyroid disorder or treatment, 40% (2191/5552) had clinical findings based on ultrasound or laboratory testing in SHIP only and 35% (1952/5552) neither a coded thyroid disorder or clinical finding nor thyroid medication. In the total study population 30% (1626/5552) received at least one TSH measurement, 6.8% (378/5552) at least one thyroid ultrasound and 2.6% (146/5552) at least one scintiscan within the past year before the study examination. Tests were performed more frequently in patients with thyroid medication and coded thyroid disorders. Hence, this group caused the highest expenditures.

**Conclusions:**

Given the high prevalence of thyroid disorders, diagnostic and monitoring tests should be used rationally with regard to costs. TSH levels should be monitored regularly in patients on thyroid medication. A consensus on monitoring frequency and iteration of monitoring of morphological thyroid disorders with TSH and ultrasound and specific guideline recommendations are needed.

**Trial registration:**

Versorgungsforschung Deutschland (VfD_17_003880).

## Background

Thyroid disorders are common in the adult German population [1–3]. Undiagnosed thyroid disorders (morphological or functional) are found in up to 75% of the population [4, 5]. Thyroid nodules are detected in 30 to 68% [4, 6, 7], most of them as incidental findings [2, 8, 9]. Some studies observed an increasing prevalence of thyroid nodules, most likely due to an improved resolution of thyroid ultrasound devices, which are now able to detect thyroid nodules of 2 to 3 mm [2, 6, 7]. The *Study of Health In Pomerania* (SHIP) found elevated serum thyroid-stimulating hormone (TSH) levels in 4% and the *Kooperative Gesundheitsforschung in der Region Augsburg* (KORA) in 14% of study participants. Supressed TSH levels were found in 5.2% (SHIP) and 2% (KORA) [4].

After an iodine fortification program was implemented in 1993 [6], goitre prevalence of the adult population in northeast Germany decreased from 35 to 30% [6] and in the age-group 11–17 years from 36 to 9% [10], but still remains common. Between 2005 and 2016, prescription rates for thyroid medications in Germany increased from 17 million [11] to 27 million per year [12]. Thyroid medications are among the ten most prescribed medications in Germany [12]. More than 75,000 thyroid surgeries are performed annually in Germany [13] and Germany has the second highest rate of thyroid surgeries in Europe (109/100,000 per year) [14].

Although clinical practice guidelines do not recommend routine screening for asymptomatic thyroid dysfunction [15], rates of thyroid function testing and diagnostic procedures increased over the last years in many countries [16, 17] and most likely also in Germany. This leads to increased diagnosis of asymptomatic patients and poses a clinical and public health problem, due to follow up, use of work force and costs.

Several guidelines focus on diagnosis and management of thyroid nodules, hypo- and hyperthyroidism and thyroid cancer [15, 18–20]. While there are data on hospital-based procedures such as surgeries, radioiodine treatment and scintiscans [21, 22], little is known about guideline implementation and the prevalence of diagnostic procedures in ambulatory care.

In a first step, this study aims to investigate the use of thyroid hormone measurements, ultrasound, scintiscan and associated costs in ambulatory care at the population level. In a second step, results will be compared with clinical guideline recommendations.

## Methods

### Design and sample

Data were derived from two independent population-based SHIP cohorts (SHIP and SHIP-TREND) including data on demography, standardised thyroid laboratory measurements and ultrasound, self-reported data from the computer-assisted interview. Ambulatory billing data (ICD-10 diagnoses (German modification of the 10th revision of the International Classification of Diseases), billing codes) from the Association of Statutory Health Insurance Physicians Mecklenburg-Vorpommern were individually linked for the period 2002–2016 with SHIP data. All participants from the second follow-up of the SHIP cohort (SHIP-2, investigation period 2008–2012; *N* = 2333, response to baseline 54%) and the SHIP-TREND cohort (investigation period 2008–2012, *N* = 4420) were eligible for inclusion [23].

**Fig. 1:**
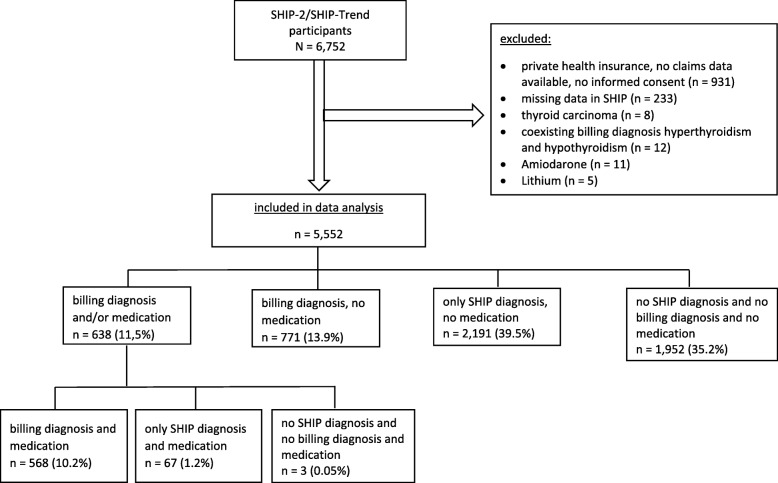
Flow chart of the study population selection

Participants without billing data or informed consent for the use of billing data, with missing clinical data on the analysed variables, with private health insurance, thyroid cancer, amiodarone or lithium treatment or concurrently coded as hyper- and hypothyroidism were excluded from analysis (*n* = 1200) (Fig. 1). We excluded participants treated with amiodarone or lithium, which affect thyroid function and -hormone metabolism, leading to biased results, because of recommended monitoring examinations.

### Data analysis

Definitions of thyroid disorders based on SHIP data and billing data are presented in Table 1. A billing diagnosis was defined as at least one relevant and confirmed ICD-10 diagnosis coded as acute or permanent within 5 years prior to the SHIP-2/SHIP-TREND examination of a participant. In order to ensure population representativeness, sampling weights were calculated and the dropout of participants between baseline and the second follow-up was considered by inverse probability weighting (ipw). To calculate ipw, a logistic regression was performed with study participation as outcome and drop-out predicting variables from baseline investigation (age, sex, school years, household income, BMI, in a partnership, diabetes, smoking status, alcohol consumption g/d, HDL cholesterol, medication intake within the last 7 days, doctor visit within the last 12 months, systolic and diastolic blood pressure, antihypertensive medication, depression, treated cancer, physical activity). The participation probability for follow-up examinations was assigned to each subject and the reciprocal of this probability was taken into account in the analysis.

**Table 1:** Definitions of thyroid disorders based on SHIP-data and claims data

Thyroid disorders	Subgroups	Clinical examination (SHIP)^a^		Billing diagnosis^b^
		laboratory value and measurements	medication (ATC codes)	ICD-10 GM
Hyperthyroidism	subclinical	TSH < 0.49 mIU/l fT4 10.10–16.50 pmol/l	no	E05.- hyperthyroidism
	clinical manifestation, untreated	TSH < 0.49 mIU/l fT4 > 16.50 pmol/l	no	
	treated	not considered	H03BB01 carbimazol H03BB02 thiamazol	
	iatrogenic	TSH < 0.49 mIU/l	H03AA.- levothyroxin /combinations	
Hypothyroidism	subclinical	TSH > 3.29 mIU/l fT4 10.10–16.50 pmol/l	no	E02.- subclinical Iodine deficiency hypothyroidism E03.- other hypothyroidism
	clinical manifestation, untreated	TSH > 3.29 mIU/l fT4 < 10.10	no	
	treated	TSH 0.49–3.29 mIU/l	H03AA.- levothyroxin / combination	
	potentially undertreated	TSH > 3.29 mIU/l	H03AA.- levothyroxin /combination	
Thyroiditis		TPO-antibody > 200 IU/ml		E06.- thyroiditis
Goitre		thyroid volume men > 25 ml women > 18 ml		E01.0 diffuse goitre due to iodine deficiency E01.1 multinodular goitre due to iodine deficiency E01.2 goitre due to iodine deficiency E03.0 congenital hypothyroidism with diffuse goitre E04.0 nontoxic diffuse goitre E04.2 nontoxic multinodular goitre E04.8 other nontoxic goitre E04.9 other not specified nontoxic goitre E05.0 hyperthyroidism with diffuse goitre E05.2 hyperthyroidism with toxic multinodular goitre E07.1 dyshormonogenetic goitre
Nodular goitre		thyroid volume men > 25 ml women > 18 ml and at least one nodule		E01.1 multinodular goitre due to iodine deficiency E04.2 nontoxic multinodular goitre E05.2 hyperthyroidism with toxic multinodular goitre
Diffuse goitre		thyroid volume men > 25 ml women > 18 ml and no nodule		E01.0 diffuse goitre due to iodine deficiency E03.0 congenital hypothyroidism with diffuse goitre E04.0 nontoxic diffuse goitre E05.0 hyperthyroidism with diffuse goitre
Thyroid nodule		ultrasound examination nodule size < 1 cm or > = 1 cm		E01.1 multinodular goitre due to iodine deficiency E04.1 nontoxic thyroid nodule E04.2 nontoxic multinodular goitre E05.1 hyperthyroidism with toxic solitary thyroid nodule E05.2 hyperthyroidism with toxic multinodular goitre D34 benign neoplasm of the thyroid
Thyroid cancer		self-reported (interview)		C73 malignancy neoplasm of the thyroid D09.3 carcinoma in situ thyroid and other endocrine gland

*ATC-Codes* Anatomical Therapeutic Chemical Classification, *ICD-10 GM* International Classification of Disease 10th Revision, German Modification, *TSH* Thyroid Stimulating Hormone, *fT3* free triiodothyronine, *fT4* free thyroxine, *TPO-antibody* Thyroid Peroxidase Antibody

^a^at the time of SHIP-2 or SHIP-TREND examination

^b^at least one relevant and confirmed ICD diagnosis coded as acute or permanent diagnosis in the billing data within 5 years prior to the SHIP-2/SHIP-TREND study entrance of the participant

The main outcomes of the study were the frequency of measurements of serum TSH level (billing code 32101), free thyroxine (fT4, billing code 32321) and free triiodothyronine (fT3, billing code 32320), TSH-receptor-antibodies (billing code 32508), thyroid peroxidase antibodies and/or thyroglobulin antibodies (billing code 32502) as well as outpatient thyroid ultrasound (billing code 33012) and scintiscan (billing code 17320) within 1 year and 3 years prior to the SHIP-2/SHIP-TREND study examinations of the participants (Additional file 1: Table S1).

A sensitivity analysis was performed to investigate the effect of including participants without billing diagnosis but with medication in the subgroup of participants *Diagnosed and/or medication* on the frequency of thyroid examinations (Fig. 1).

In a subgroup analysis, multinomial logistic regression models were used to assess the association with age, sex, thyroid medication intake and Charlson-Comorbidity-Index (CCI) with the frequency of TSH measurements and ultrasound examinations. CCI was calculated based on billing diagnoses from 2002 to 2006 [24]. The outcomes were grouped in frequencies of 0, 1 and > 1. Multicollinearity, occurring when two or more predictor variables are moderately or highly correlated, was tested using Pearson correlation. We did not find any variables with moderate or high correlation (cut-off 0.4). Corresponding costs were calculated using quarterly billing codes for the respective study period at the time of the study, therefore considering changes in costs over time. The analysis was performed using SAS software version 9.4 (SAS Institute Inc., Cary, NC, USA).

## Results

### Participant characteristics

A total of 5552 participants (47% male, median age 55) were included in the analysis (Fig. 1, Table 2). 25% (1409/5552) of the participants had a billing diagnosis or treatment, 40% (2191/5552) had clinical findings based on ultrasound or laboratory testing in SHIP only and 35% (1952/5552) had neither a coded thyroid disorder or clinical finding nor thyroid medication. All thyroid disorders, but especially goitre and thyroid nodules were more frequently found in SHIP compared to billing diagnosis. Thyroid medication, including levothyroxine and combinations, thiamazole and iodide were prescribed in 11.5%.

**Table 2:** Patient characteristics (*N* = 5552)

	Number (N)	Percent (%)	95% CI
Sex (male)	2602	46.9	45.5–48.2
Age			
≤ 30	327	5.9	5.3–6.5
> 30–50	1876	33.8	32.5–35.0
> 50–80	3237	58.3	57.0–59.6
> 80	112	2.0	1.7–2.4
Pregnant women	8	0.1	0.04–0.2
BMI > 30	1825	32.8	31.6–34.1
Thyroid medication	638	11.5	10.6–12.3
CCI			
0	3049	54.9	53.6–56-2
1	1390	25.0	23.9–26.2
2–5	1038	18.7	17.7–19.7
6–12	36	0.7	0.4–0.9
SHIP diagnosis^a, c^			
Goitre	1815	32.7	31.5–33.9
Hyperthyroidism	510	9.2	8.4–9.9
Hypothyroidism	611	11.1	10.2–11.8
Thyroid nodule	2197	39.6	38.3–40.9
Thyroiditis	234	4.2	3.7–4.7
Billing diagnosis^b, c^			
Goitre	1057	19.0	18.0–20.1
Hyperthyroidism	230	4.1	3.6–4.7
Hypothyroidism	320	5.8	5.2–6.4
Thyroid nodule	500	9.0	8.2–9.8
Thyroiditis	166	2.99	2.5–3.4

^a^clinical findings based on ultrasound or laboratory testing in SHIP, regardless of billing diagnosis

^b^coded thyroid disorder in billing data, regardless of SHIP findings

^c^one patient can have more than one diagnosis

### Management and monitoring

#### Descriptive statistics

In the total study population, at least one TSH measurement was performed in 30% (1626/5552), at least one ultrasound in 6.8% (378/5552) and at least one scintiscan in 2.6% (146/5552) within the past year before the SHIP examination. Participants with billing diagnosis and/or medication received the highest number of examinations followed by those with billing diagnoses and no medication. In participants with billing diagnosis and/or medication, 60% received at least one TSH measurement within 1 year (Table 3) and 84% within 3 years before SHIP examination (Table 4). In those with billing diagnosis and no medication, 43% received at least one TSH measurement within 1 year and 74% within 3 years before examination. Participants with thyroid disorder diagnosed only in SHIP and participants with no thyroid disorder and no medication received at least one TSH measurement in 23 and 22% of cases 1 year before the SHIP examination and in 46 and 40% of cases 3 years before examination. At least one thyroid ultrasound scan was performed in up to 22% of participants with billing diagnosis and/or medication 1 year before examination, and in up to 47% 3 years before examination.

**Table 3:**
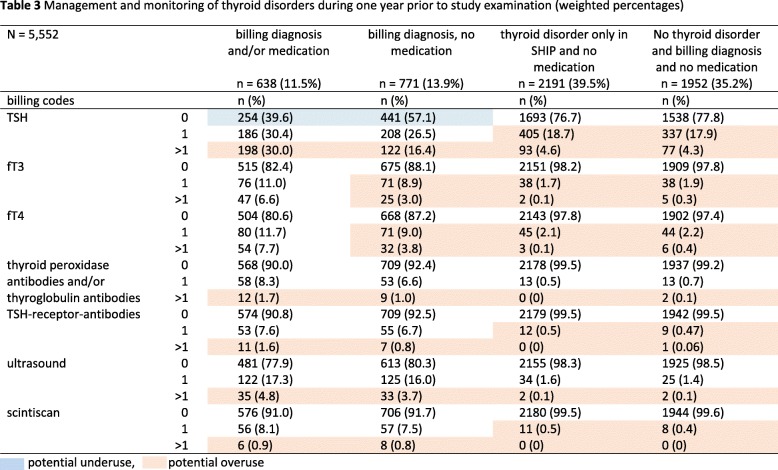
Management and monitoring of thyroid disorders during 1 year prior to study examination (weighted percentages)

potential underuse,  potential overuse

**Table 4:**
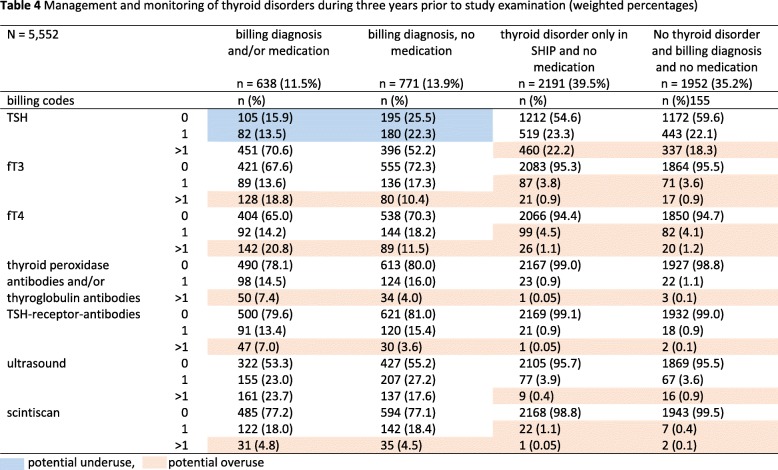
Management and monitoring of thyroid disorders during 3 years prior to study examination (weighted percentages)

potential underuse,  potential overuse

Participants with a thyroid disorder diagnosed only in SHIP and participants with no thyroid disorder and no medication received at least one ultrasound scan in in up to 2% 1 year before examination and in up to 5% 3 years before examination. At least one scintiscan was carried out in up to 9% of participants with a billing diagnosis 1 year before examination and in up to 23% 3 years before examination.

### Multivariate analysis

In the total study population, the odds to receive more than one TSH measurement within 1 year prior to SHIP examination was increased in participants taking thyroid medication, participants with a higher CCI and women (Table 5). Age was weakly associated with the number of TSH measurements. Participants taking medication and women were more likely to receive more than one thyroid ultrasound examination (Table 5). In participants with a billing diagnosis and / or medication, medication and female sex were associated with more than one TSH measurement. Individuals with a high CCI had a higher risk to have more than one TSH measurement compared to individuals with a low CCI. Only the CCI was associated with the number of thyroid ultrasound examinations.

**Table 5:** Crude and adjusted multinomial logistic regression models with TSH-measurement frequency and ultrasound frequency within 1 year prior to SHIP examination as outcome variables in all participants and only participants with billing diagnosis and/or medication (weighted)

Determinants	TSH measurement during 1 year prior to SHIP examination							
	All participants *N* = 5552				Participants with a billing code and/ or medication *n* = 1409			
	Crude OR (95% CI)		Adjusted OR (95% CI)		Crude OR (95% CI)		Adjusted OR (95% CI)	
	1 TSH	> 1 TSH	1 TSH	> 1 TSH	1 TSH	> 1 TSH	1 TSH	> 1 TSH
Medication	**2.88** (2.44–3.39)	**8.66** (7.23–10.37)	**2.32** (1.96–2.76)	**6.29** (5.19–7.61)	**1.65** (1.35–2.02)	**2.64** (2.11–3.29)	**1.66** (1.34–2.04)	**2.48** (1.98–3.11)
Sex (female)	**1.29** (1.16–1.44)	**2.21** (1.88–2.59)	**1.29** (1.15–1.44)	**1.83** (1.53–2.18)	0.94 (0.76–1.17)	**1.45** (1.13–1.86)	0.93 (0.74–1.16)	**1.36** (1.04–1.76)
Age (continuous)	**1.03** (1.03–1.04)	**1.04** (1.03–1.04)	**1.03** (1.02–1.03)	**1.02** (1.01–1.03)	**1.02** (1.01–1.03)	1.01 (1.00–1.02)	**1.02** (1.01–1.03)	1.00 (0.99–1.02)
CCI^a^	**1.22** (1.17–1.26)	**1.37** (1.31–1.43)	**1.08** (1.04–1.13)	**1.26** (1.19–1.32)	1.05 (0.98–1.13)	**1.16** (1.09–1.24)	0.99 (0.92–1.06)	**1.15** (1.06–1.23)
	Thyroid ultrasound during 1 year prior to SHIP examination							
	All participants *N* = 5552				Participants with a billing code and/or medication *n* = 1409			
	Crude OR (95% CI)		Adjusted OR (95% CI)		Crude OR (95% CI)		Adjusted OR (95% CI)	
	1 ultrasound	> 1 ultrasound	1 Ultrasound	> 1 Ultrasound	1 Ultrasound	> 1 Ultrasound	1 Ultrasound	> 1 Ultrasound
Medication	**5.39** (4.39–6.63)	**8.48** (5.65–12.73)	**4.24** (3.41–5.29)	**6.79** (4.41–10.48)	1.11 (0.89–1.39)	1.35 (0.88–2.06)	1.14 (0.90–1.44)	1.35 (0.88–2.08)
Sex (female)	**2.02** (1.65–2.47)	**4.93** (2.89–8.39)	**1.58** (1.27–1.96)	**3.20** (1.84–5.54)	0.92 (0.72–1.18)	**2.03** (1.16–3.58)	0.88 (0.67–1.13)	1.66 (0.93–2.96)
Age (continuous)	**1.02** (1.01–1.03)	1.00 (0.98–1.02)	**1.02** (1.01–1.02)	1.00 (0.98–1.02)	0.99 (0.98–1.00)	**0.97** (0.96–0.99)	0.99 (0.98–1.01)	0.98 (0.97–1.01)
CCI^a^	**1.12** (1.06–1.18)	0.86 (0.72–1.02)	1.03 (0.96–1.09)	**0.81** (0.67–0.98)	0.98 (0.91–1.06)	**0.69** (0.56–0.85)	0.99 (0.91–1.07)	**0.75** (0.61–0.94)

^a^*CCI* Charlson-Comorbidity-Index, significant results have been marked in boldface type

### Sensitivity analysis

The group of participants with billing diagnosis and/or medication (*n* = 638; 11.5%) includes participants with medication and thyroid disorders diagnosed only in SHIP (67 participants) and participants with medication intake and no SHIP nor billing diagnosis (3 participants) (Fig. 1). This may have resulted in biased results. Hence, frequencies of the examinations were re-calculated after excluding these 70 participants. The proportion of participants with at least one measurement of TSH 1 year before examination increased by 4%, of fT3 and fT4 by 1.7%, ultrasound by 2.4%, scintiscan by 0.9%, and measurements of thyroid peroxidase antibodies and/or thyroglobulin antibodies by 1.2% and TSH-receptor-antibodies by 1%.

### Expenditures

Table 6 gives an overview of thyroid associated expenditures 1 year prior to study examination. The highest expenditures were caused by GP consultations, scintiscan and ultrasound. Participants with billing diagnosis and/or medication caused the highest expenditures (335.55 € per person), followed by participants with billing diagnosis and no medication (298.39 € per person) and participants with thyroid disorder only in SHIP and no medication (228.14 € per person). Participants without thyroid disorder and billing diagnosis and no medication caused the lowest expenditures.

**Table 6:** Expenditures 1 year before SHIP examination

	Billing diagnosis and/or medication *n* = 638		Billing diagnosis, no medication *n* = 771		Thyroid disorder only in SHIP and no medication *n* = 2191		No thyroid disorder and billing diagnosis and no medication *n* = 1952	
	Costs (€)	Costs per person (€)	Costs (€)	Costs per person (€)	Costs (€)	Costs per person (€)	Costs (€)	Costs per person (€)
TSH (3.00 €)^a^	2349.00	3.68	1563.00	2.03	1836.00	0.84	1554.00	0.80
ultrasound (23.00€/24.00€)^a^	5013.00	7.86	4863.00	6.31	911.00	0.42	719.00	0.37
scintiscan (114.50 €)^a^	7900.50	12.38	8459.00	10.97	1257.50	0.57	916.00	0.47
fT3 (3.70 €/4.10 €)^a^	786.70	1.23	499.10	0.65	165.10	0.08	184.50	0.09
fT4 (3.70 €/4.10 €)^a^	903.40	1.42	572.20	0.74	198.00	0.09	214.50	0.11
TSH-receptor-antibodies (10.30 €/ 11.20€)^a^	822.30	1.28	740.80	0.96	126.30	0.06	114.20	0.06
thyroid peroxidase antibodies (7.50 €/8.70€)^a^	662.70	1.04	581.40	0.75	101.10	0.05	132.30	0.07
endocrinological consultation (61.50 €/63.50 €)^a, b^	1877.00	2.94	1573.50	2.04	63.50	0.03	317.50	0.16
GP consultation^a, b, c^	193,768.00	303.71	211,205.00	273.94	496,764.50	226.73	405,062.50	207.51
total costs	214,082.60	335.55	230,057.00	298.39	501,423.00	228.14	409,214.50	209.64

^a^costs were calculated considering the date of the measurements or consultations

^b^yearly expenditures with maximum one billing code per quarter

^c^costs considering /consultation date: 83.50 € / 88.00 € / 90.00 € / 102.00 €; not included are lump sum payment for chronically ill patients for the study period 2005–2007 (9.00 €/ 14.50 €/ 22.50€/ 32.00€)

## Discussion

This study aimed to investigate the use of thyroid hormone measurements, thyroid ultrasound, thyroid scintiscan and associated costs in ambulatory care at population level. In the total study population, 30% received at least one TSH measurement, 6.8% at least one thyroid ultrasound and 2.6% at least one scintiscan during 1 year before study examination. Laboratory tests and examinations were performed most frequently in those with coded thyroid disorders and thyroid medication and those with coded thyroid disorders without medication intake. These groups also caused the highest expenditures. Participants without thyroid disorders, billing diagnosis and no medication and participants with thyroid disorder only in SHIP not taking medication were less frequently examined at comparable rates.

### Thyroid laboratory tests

There are few data on the frequency / use of thyroid laboratory testing and diagnostic procedures. One German study focuses on billing data analyses of diagnostic testing and procedures in newly diagnosed patients with thyroid nodules [21]. In this study, in the year before diagnosis, thyroid ultrasound was performed in 86% (uninodular goitre) and 75% (multinodular goitre), TSH measurements in up to 78% and scintiscans in up to 44% of patients without subsequent surgery. In this patient group, within 2 years after being newly diagnosed, ultrasound was performed in 28% (uninodular goitre) and in 24% (multinodular goitre), TSH measurements in up to 48% and scintiscans in up to 11% [21]. Data indicate that diagnostic tests are performed more frequently than monitoring tests and patients with subsequent surgery are tested more frequently. The results for the time period after diagnosis in patients without surgery are comparable to the observed rates in our study 1 year before examination but markedly lower than those observed 3 years before examination, although authors included only patients with thyroid nodules [21].

In the UK, thyroid laboratory tests including fT3, fT4 and TSH cumulated to 10 million annually in 2003, costing approximately £ 30 million each year [25, 26]. A report on regional variations of diagnostic services in the UK found a high variation in GP ordered thyroid function tests with annual rates of 6 to 356 per 1000 practice patients for TSH (5 to 257 per 1000 for fT4, 0.04 to 7.0 per 1000 for fT3) [27]. Vaidya et al. [17] reported an up to six-fold variation in the annual rate of ordered TSH tests per 1000 practice patients in Southwest England. Authors found most of the variation to be due to heterogeneity across practices and only 24% of the variation was accounted for by hypothyroidism and socio-economic deprivation. In our study, TSH measurement rates were 1.7 times higher than the highest reported rate (438/1000), fT4 measurement rates were within the reported range (89/1000) and fT3 was measured at a 10-fold higher rate than the reported one (89/1000).

Although our study also includes laboratory tests performed by primary care specialists, our data suggests overuse especially in SHIP participants without thyroid medication. In this participant group, TSH measurements were performed in up to 43% 1 year and in up to 75% 3 years prior to SHIP examination. Because the test date of the investigated procedures and billing codes in our study is only available on a quarterly basis and no starting date for the medication was available, it was not possible to distinguish between diagnostic and monitoring tests. The proportion of participants with one TSH test or more was higher in those with diagnosed thyroid disorder with medication vs. no medication, suggesting a possible higher demand for monitoring with medication / changes in medication. However, we suggest that the high rate of TSH testing in patients with diagnosed thyroid disease *without* treatment indication reflects the lack of specific guideline recommendations for monitoring. Due to recommended minimum intervals of 6 to 8 weeks between TSH measurements, a definite overuse could be defined as more than six measurements per year. In our study the proportion of participants with diagnosed thyroid disorder taking medication with more than six TSH measurements was 1.1% (7/638).

According to guidelines, TSH rather than fT4/fT3 should be used in patients with suspected hypo- and hyperthyroidism and for monitoring purposes [15]. Our data show markedly lower proportions of fT4 / fT3 testing than TSH testing in all investigated groups, which is in line with the guidelines. Since we have no information on possible clinical symptoms or stability of TSH levels we cannot investigate overuse. However, our data suggest that fT4 and fT3 were tested at the same rate, despite fT4 is recommended as sufficient to distinguish between overt and subclinical hypothyroidism. In participants *without* diagnosed thyroid disorders and thyroid medication, there was no clinically significant difference in the rate of thyroid hormone testing between those with and without clinical study findings. These patient groups may include patients with coded suspected thyroid causes (diagnoses) and / or general symptoms, as well as those screened for thyroid disease without symptoms. We assume, that these numbers reflect the frequent ambulatory diagnostic testing for hypothyroidism in connection with unspecific symptoms including tiredness, weight-gain and low energy. Moreover, a possible overuse of TSH testing might be attributed to a lack of specific guideline recommendations. Since no ambulatory laboratory values are available, it is not possible to distinguish repeated testing because of altered TSH levels on previous tests as recommended by the guidelines.

Our results suggest that despite overuse of thyroid hormone testing there is a possible underuse in patients with diagnosed thyroid disorders taking thyroid medication. In this group, 40% did not receive a monitoring TSH test within 1 year, and 16% within 3 years prior to SHIP examination [15].

Thyroid antibody testing (thyroid peroxidase antibodies and/or thyroglobulin antibodies and TSH-receptor-antibodies) was more frequent in participants with diagnosed thyroid disorders independent of medication use. Since we cannot distinguish between diagnostic and monitoring tests, it is unclear, if our results are suggestive of test overuse.

Ultrasound is a recommended diagnostic procedure for thyroid nodules and goitre and for follow-up depending on sonographic features, growth and risk for malignancy. For this study, we did not include data on sonographic thyroid characteristics and data on clinical complaints are not available. However, an overuse of ultrasound in the monitoring of thyroid disorders cannot be ruled out. Scintiscans were performed at similar rates in participants with diagnosed thyroid disorder with and without medication and at very low rates in patients without thyroid disorder and / or without a billing code.

On multivariate analysis, thyroid medication, female sex and CCI were associated with the number of TSH measurements and thyroid ultrasound examinations, suggesting use for monitoring purposes and a possible higher rate of laboratory testing in persons with more frequent consultations.

### Expenditures

Expenditures for the investigated tests in participants with thyroid disease and medication were 125 Euros per person more than in patients without thyroid disease and without medication. However, total scintiscans expenditures were highest in the group with diagnosed thyroid disease without medication, suggesting use for diagnostic purposes. The higher costs in diagnosed and / or treated patients for thyroid hormone testing might partly be due to repeated measurements performed in specialist care as part of a routine follow-up examination despite test results already available in the GP practice.

Our data shows that 40% of the study population had an undiagnosed thyroid disorder (Tables 3 and 4, ‘*thyroid disorder only in SHIP and no medication’)*. We hypothesise, that these subjects could be categorised in the same group as *participants with billing diagnosis and no medication*. If both groups were tested at similar rates, total expenditures would increase by additional 152,350 Euro for our study population including consultation expenditures. If only thyroid testing costs were included in patients with physician-diagnosed thyroid disorders or thyroid disorders diagnosed only in SHIP, costs would amount to additional 44,500 Euro for 1 year prior to study. Assuming the same proportion of undiagnosed thyroid changes in the general population (40%, *n* = 557,872), expenditures for thyroid testing excluding consultation costs would amount to additional 12.5 million Euro and including consultation costs to 166.5 million Euro in Mecklenburg-Vorpommern with an adult population of 1.39 million for 1 year. Thyroid testing overuse additionally leads to increased indirect costs including medical resource use (consultations and laboratory capacity) and may increase distress to patients.

### Strengths and limitations

This is the first population-based study investigating rates of thyroid testing in Germany. We used inverse probability weighting to account for selective drop out during the study period. The interpretation of our results is limited by the lack of clinical and laboratory data for ambulatory care. Therefore, it was not possible to distinguish between diagnostic and monitoring tests and to define cases needing a more frequent monitoring than recommended by guidelines. We were not able to investigate thyroid fine needle aspiration biopsy, because the billing code does not distinguish between different biopsies. Similarly, general practitioner consultations could not be attributed to thyroid disorders. Data on expenditures do not include costs for medication and indirect costs as staff, transport, controls and device maintenance. We had no data on inpatient treatment of study participants.

## Conclusion

Our data indicates potential overuse of TSH, fT3 and fT4 testing especially in patients with diagnosed thyroid disorders not taking thyroid medication. Thyroid ultrasound is potentially overused in patients with diagnosed and / or treated thyroid disorders. However, in some participants with diagnosed thyroid disorders taking medication TSH monitoring is still underused.

Given the frequency of patients with thyroid disorders, diagnostic and monitoring tests should be used rationally with regard to costs. TSH levels should be monitored regularly in patients on thyroid medication. A consensus on monitoring frequency and iteration of monitoring of morphological thyroid disorders with TSH and ultrasound and specific guideline recommendations are needed.

## Supplementary information

**Additional file 1: Table S1.** Diagnostic procedures and their corresponding billing codes.

## Data Availability

Billing Data cannot be shared publicly due to legal restrictions regarding claims data according to SGB XI. Data from the SHIP-Study is available on reasonable request according the bylaws of the research association of the community medicine https://www.fvcm.med.uni-greifswald.de/dd_service/data_use_intro.php.

## Abbreviations

BMI: Body Mass Index
CCI: Charlson-Comorbidity-Index
fT3: Free triiodothyronine
fT4: Free thyroxine
HDL: High-Density Lipoprotein
ICD-10: International Classification of Diseases, 10^th^ Revision
ipw: Inverse probability weights
KORA: Kooperative Gesundheitsforschung in der Region Augsburg
SHIP: Study of Health in Pomerania
TSH: Thyroid-stimulating hormone
