# Supplementary material for: Frequency of thyroid function tests and examinations in participants of a population-based study
Source: BMC Health Serv Res. 2020 Jan 30;20:70. doi: 10.1186/s12913-020-4910-7 (PMC6993310; doi:10.1186/s12913-020-4910-7)
Supplement: Supplementary file 1 — Additional file 1: Table S1. Diagnostic procedures and their corresponding billing codes. [file 12913_2020_4910_MOESM1_ESM.docx]

Table S1: Diagnostic procedures and their corresponding billing codes

| diagnostic procedure | corresponding billing code |
| --- | --- |
| measurement of |  |
| serum TSH level | 32101 |
| free thyroxine (fT4) | 32321 |
| free triiodothyronine (fT3) | 32320 |
| TSH-receptor-antibodies | 32508 |
| thyroid peroxidase antibodies and/or thyroglobulin antibodies | 32502 |
| thyroid ultrasound | 33012 |
| scintiscan | 17320 |
